# The association between socioeconomic disparities and left ventricular hypertrophy in chronic kidney disease: results from the KoreaN Cohort Study for Outcomes in Patients With Chronic Kidney Disease (KNOW-CKD)

**DOI:** 10.1186/s12882-018-1005-3

**Published:** 2018-08-16

**Authors:** Eunjeong Kang, Joongyub Lee, Hyo Jin Kim, Miyeun Han, Woo Wan Kim, Kyu-Beck Lee, Suah Sung, Tae-Hyun Yoo, Wookyung Chung, Curie Ahn, Kook-Hwan Oh

**Affiliations:** 1 Department of Internal Medicine, Seoul National University College of Medicine, Seoul, Korea; 2 School of Medicine, Inha University, Incheon, Korea; 3 Department of Prevention and Management, Inha University Hospital, Incheon, Korea; 4 Department of Internal Medicine, Dongguk University Gyeongju Hospital, Gyeongju, Korea; 5 Department of Internal Medicine, Pusan National University Hospital, Pusan, Korea; 6 Department of Internal Medicine, Chonnam National University Medical School, Gwangju, Korea; 7 Division of Nephrology, Kangbuk Samsung Hospital, Sungkyunkwan University School of Medicine, Seoul, Korea; 8 Department of Internal Medicine, Nowon Eulji Medical Center, Eulji University, Seoul, Korea; 9 Department of Internal Medicine, Yonsei University College of Medicine, Seoul, Korea; 10 Department of Internal Medicine, Gachon University Gil Medical Center, Gachon University School of Medicine, Incheon, Korea

**Keywords:** Chronic kidney disease, Left ventricular hypertrophy, Education, Income, Socioeconomic status

## Abstract

**Background:**

Left ventricular hypertrophy (LVH) is one of the risk factors for cardiovascular (CV) disease and mortality. However, the relationship between socioeconomic status (SES) and LVH in chronic kidney disease remains unclear.

**Methods:**

Data were collected from the KoreaN Cohort Study for Outcome in Patients With Chronic Kidney Disease (KNOW-CKD, NCT01630486 at http://www.clinicaltrials.gov). Subjects with CKD and aged ≥50 were included. SES was characterized based on monthly income and educational attainment, each of which was divided into three strata. LVH was defined as LV mass/height^2.7^ ≥ 47 g/m^2.7^ in female and ≥ 50 g/m^2.7^ in male. Age, sex, diabetes, CKD stage, body mass index, blood pressure and physical activity were included as covariates.

**Results:**

A total of 1361 patients were included. Mean age was 60.9 ± 6.9 years, and 63.2% were men. Higher education level was associated with higher monthly income (*P* for trend < 0.001). The lowest education level was independently associated with LVH (lower than high school, adjusted odds ratio [OR] 1.485, 95% CI 1.069–2.063, *P* = 0.018; completed high school, adjusted OR 1.150, 95% confidence interval [CI] 0.834–1.584, *P* = 0.394; highest education level as the reference). Monthly income level was marginally associated with LVH after adjusting for covariates ($1500-4500, adjusted OR 1.230, 95% CI 0.866–1.748, *P* = 0.247; < $1500, adjusted OR 1.471, 95% CI 1.002–2.158, *P* = 0.049; > $4500; reference).

**Conclusions:**

In the CKD population, lower SES, defined by educational attainment and low income level exhibited a significant association with LVH, respectively. Longitudinal follow-up will reveal whether lower SES is associated with poor CKD outcomes.

## Backgrounds

Socioeconomic status (SES) is an important and strong predictor of morbidity and mortality. [1] Generally, education level, occupation, race, housing, social support and income are key components to be evaluated as SES. Multiple determinants of health care level vary with SES levels, including risk of all-cause mortality, [2] cardiovascular diseases, [3–5] diabetes mellitus, [5, 6] cancer, [7, 8] and chronic kidney disease (CKD) [5, 9]. The reasons behind this phenomenon have been suggested as follows: educational attainment and income levels contribute to a complex set of socio-economic determinants, including insurance, transportation, stress, housing quality and access to health care [10]. Such determinants may interact and combine to affect the health outcomes in an interconnected mechanism. Because SES appears to affect CKD patients in a similar way as it does the general population, it is important to clarify the health-related risk factors in CKD influenced by SES.

Worldwide, cardiovascular diseases are the leading cause of death among patients with CKD and end-stage renal disease (ESRD). Cardiovascular mortality is the primary cause of death in CKD patients in Korea, accounting for the 39% of mortality cases in peritoneal dialysis and 36% in hemodialysis patients [11]. Thus, most physicians make every effort to prevent a cardiovascular event and control its risk factors, such as lipid levels and anemia. Above all, left ventricular hypertrophy (LVH) causes decreased diastolic compliance and leads to ischemic cardiomyopathy, even in the absence of coronary artery disease [12]. More specifically, in a cohort of patients starting dialysis therapy, cardiac enlargement and decreased systolic function exhibited a relationship with ischemic heart disease and cardiac failure [13].

Meanwhile, in a previous study, Carlos et al. [14] reported that lower SES is an independent risk factor for increased left ventricular mass among hypertensive and normotensive African Americans. However, the relationship between SES and risk factors of cardiovascular mortality, including left ventricular hypertrophy (LVH), are less well known among CKD patients.

Since some social determinants of health are modifiable through education and governmental health policies, investigating the influence of SES on the outcome of CKD is crucial. Therefore, we investigated the association between LVH, the representative risk factor for CV mortality in CKD and socioeconomic status, evaluated by educational attainment and monthly income level, among participants in the KoreaN cohort study for Outcome in patients With Chronic Kidney Disease (KNOW-CKD).

## Methods

### Study population

**Fig. 1:**
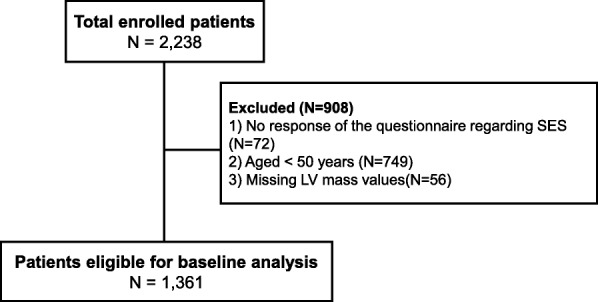
Study Flow. Abbreviations: SES, socioeconomic status; LV, left ventricular

Participants in the KNOW-CKD, a Korean multicenter prospective cohort study that enrolled subjects with CKD from stage 1 to 5 (predialysis) from June 30, 2011 to January 29, 2016, were included in this cross-sectional analysis. The detailed design and methods of the KNOW-CKD have been previously published elsewhere [15]. In total, 2238 participants were enrolled in the KNOW-CKD study. Among them, we excluded individuals who did not respond to the questionnaire regarding SES, no measured left ventricular (LV) mass, and who were aged < 50 years (Fig. 1). Finally, 1330 subjects were included in the analyses.

### Variable measurements

Demographics, and clinical and laboratory values at enrollment were extracted from an electronic data management system (http://www.phactaX.org). The estimated glomerular filtration rate (eGFR) was estimated using the Chronic Kidney Disease Epidemiology Collaboration (CKD-EPI) equation using creatinine [16]. Resting blood pressure was measured with mercury sphygmomanometers and cuffs of appropriate size three times for average blood pressure. Hypertension (HTN) was defined as a blood pressure recording ≥140/90 mmHg, a self-reported history of hypertension, or use of antihypertensive agents. Diabetes (DM) was defined by self-reporting or use of hypoglycemic medications. Physical activity was quantified by the International Physical Activity Questionnaire. Subjects were categorized by total Metabolic Equivalent of Task (MET) - minutes/week; “high” was defined as ≥3000 METs-minutes/week, “moderate” as 600–2999 METs-minutes/week and “low” as < 600 METs-minutes/week. Anemia was defined as hemoglobin < 13 g/dL for males, or < 12 g/dL for females. Two-dimensional echocardiography was conducted to measure cardiac parameters. LV mass was calculated by 0.8 x {1.04[(LVIDd + PWTd + SWTd)^3^ - (LVIDd)^3^]} + 0.6 g, where PWTd and SWTd are posterior wall thickness at end diastole and septal wall thickness at end diastole, respectively [17]. Left ventricular hypertrophy (LVH) was defined as LV mass/height^2.7^ ≥ 47 g/m^2.7^ in female and ≥ 50 g/m^2.7^ in male, [17, 18] because LV mass indexed to body surface area is problematic in that weight is affected by volume overload in CKD [19].

### Evaluation of socioeconomic status

The questionnaire used in the KNOW-CKD Study followed those of the Korea National Health and Nutrition Examination Survey (KNHANES). The KNAHES classified the monthly income of Korean population into three intervals, based on the average household income of the contemporary Koreans. Information on educational attainment and monthly income level, which were captured from patient-reported questionnaire, was used as indicators of SES. With regard to educational attainment, the patients were asked about the level at which their formal school education was completed. Educational attainment was classified into three levels: “less than high school” included those who never went to high school or who completed only part of high school, “completed high school” included those who had graduated high school but not completed college, “college degree or beyond” included those who had completed college or a higher degree. Monthly income level was classified into three levels: less than $ 1500, $ 1500 to $ 4500, and over $ 4500 per month.

### Statistical analysis

Continuous variables are presented as a mean ± standard deviation. Proportions were used for categorical variables, including age groups, sex, CKD stages, comorbidities (e.g., diabetes mellitus). We used one-way analysis of variance for comparison of continuous variables and the χ^2^ test for categorical variables. Statistical significance was determined at *P* < 0.05 using two-sided tests. We conducted logistic regression to evaluate the association between LVH and SES, which is categorized into three education or monthly income levels. We checked the Hosmer-Lemeshow goodness of fit test for determining whether multivariable models are fit of data. Statistical analyses were carried out using the SPSS software package, version 22.0 (IBM Corporation, Armonk, NY, USA).

### Ethics statement

The study protocol was approved by the Institutional Review Board at each participating clinical center — i.e., Seoul National University Hospital (1104–089-359), Seoul National University Bundang Hospital (B-1106/129–008), Yonsei University Severance Hospital (4–2011-0163), Kangbuk Samsung Medical Center (2011–01-076), Seoul St. Mary’s Hospital (KC11OIMI0441), Gil Hospital (GIRBA2553), Eulji General Hospital (201105–01), Chonnam National University Hospital (CNUH-2011-092), and Pusan Paik Hospital (11–091) in 2011. This study was conducted in accordance with the principles of the Declaration of Helsinki.

## Results

### Baseline characteristics of study participants

The baseline characteristics of the study subjects are shown in Table 1. Mean age was 60.9 ± 6.9 years, and 860 (63.2%) were men. Mean eGFR was 45.5 ± 25.1 mL/min/1.73 m^2^ and the number of patients with a CKD stage G3a, G3b, G4, and G5 were 226 (16.6%), 250 (18.4%), 322 (23.7%), 352 (25.9%) and 1023 (75.1%), respectively. Diabetic nephropathy was the most common cause of CKD (31.8%), followed by glomerulonephritis (25.3%), hypertensive nephropathy (24.2%), and polycystic kidney disease (10.4%). Subjects with DM and HTN comprised 42.0% and 98.0% of the study participants, respectively. Anemia was prevalent in 662 (49.1%) subjects and 119 (8.7%) subjects received erythropoiesis-stimulating agents (ESAs).

**Table 1:** Baseline Characteristics according to Educational attainment

N	Total	Educational attainment			*P*	*P* for trend
		College or beyond	Completed High school	Lower than high school		
	1361	437	467	457		
Age (year, mean ± SD)	60.9 ± 6.9	60.3 ± 7.1	60.0 ± 6.9	62.4 ± 6.6	< 0.001	< 0.001
Male (N, %)	860 (63.2)	361 (82.6)	299 (64.0)	200 (43.8)	< 0.001	< 0.001
LVH (N, %)	413 (30.3)	101 (23.1)	132 (28.3)	180 (39.4)	< 0.001	< 0.001
eGFR (mL/min/1.73m^2^)	45.5 ± 25.1	47.1 ± 23.3	46.7 ± 26.6	42.7 ± 25.0	0.001^a^	0.008
BMI (kg/m^2^, mean ± SD)	24.6 ± 3.1	24.6 ± 2.9	24.4 ± 3.2	24.9 ± 3.2	0.049	0.155
CKD stage (N, %)					< 0.001	< 0.001
Stage G1	112 (8.2)	27 (6.2)	50 (10.7)	35 (7.7)		
Stage G2	226 (16.6)	89 (20.3)	72 (15.4)	65 (14.2)		
Stage G3a	250 (18.4)	107 (24.5)	83 (17.8)	60 (13.1)		
Stage G3b	322 (23.7)	90 (20.6)	112 (24.0)	120 (26.3)		
Stage G4	352 (25.9)	101 (23.1)	114 (24.4)	137 (30.0)		
Stage G5	99 (7.3)	23 (5.3)	36 (7.7)	40 (8.8)		
Underlying renal disease (N, %)					0.042	0.049
Diabetic nephropathy	429 (31.8)	130 (29.9)	142 (30.7)	157 (34.7)		
Hypertensive nephropathy	327 (24.2)	109 (25.1)	105 (22.7)	113 (25.0)		
Glomerulonephritis	341 (25.3)	104 (23.9)	136 (29.4)	101 (22.3)		
Polycystic kidney disease	140 (10.4)	60 (13.8)	42 (9.1)	38 (8.4)		
Diabetes mellitus (N, %)	571 (42.0)	168 (38.4)	189 (40.5)	214 (46.8)	0.022	0.019
^b^Hemoglobin A1c (%, mean ± SD)	6.9 ± 1.4	6.7 ± 1.2	6.9 ± 1.3	7.1 ± 1.5	0.005^a^	0.001
Hypertension (N, %)	1334 (98.0)	430 (98.4)	457 (97.9)	447 (97.8)	0.673	0.635
Systolic blood pressure (mmHg, mean ± SD)	128.84 ± 16.7	127.8 ± 15.2	129.1 ± 17.3	129.5 ± 17.5	0.539^a^	0.138
Diastolic blood pressure (mmHg, mean ± SD)	75.9 ± 11.2	76.3 ± 10.7	76.6 ± 10.9	74.9 ± 11.8	0.071	0.068
Monthly income (N, %)					< 0.001	< 0.001
> $ 4500	269 (19.8)	163 (37.3)	73 (15.6)	33 (7.2)		
$ 1500 to 4500	701 (51.5)	214 (49.0)	279 (59.7)	208 (45.5)		
< $ 1500	391 (28.7)	60 (13.7)	115 (24.6)	216 (47.3)		
Physical activity (N, %)					< 0.001	< 0.001
High	287 (21.1)	95 (21.7)	112 (24.0)	80 (17.5)		
Moderate	536 (39.4)	201 (46.0)	170 (36.4)	165 (36.1)		
Low	538 (39.5)	141 (32.3)	185 (39.6)	212 (46.4)		
Anemia (N, %)	662 (49.1)	178 (41.2)	225 (48.8)	259 (57.0)	< 0.001	< 0.001
ESA (N, %)	119 (8.7)	30 (6.9)	33 (7.1)	56 (12.3)	0.005	0.006
Serum laboratory values						
Hemoglobin (g/dL, mean ± SD)	12.6 ± 2.0	13.2 ± 2.0	12.6 ± 1.9	12.0 ± 1.92	< 0.001	< 0.001
Creatinine (mg/dL, mean ± SD)	1.9 ± 1.1	1.9 ± 1.1	1.9 ± 1.1	1.9 ± 1.1	0.986	0.869
Albumin (g/dL, mean ± SD)	4.2 ± 0.4	4.2 ± 0.4	4.2 ± 0.4	4.1 ± 0.4	0.014	0.003
Calcium (mg/dL, mean ± SD)	9.1 ± 0.5	9.1 ± 0.5	9.1 ± 0.5	9.1 ± 0.5	0.413	0.206
Phosphorus (mg/dL, mean ± SD)	3.7 ± 0.7	3.6 ± 0.6	3.7 ± 0.7	3.9 ± 0.7	< 0.001	< 0.001
Total cholesterol (mg/dL, mean ± SD)	170.7 ± 38.2	168.8 ± 36.8	170.6 ± 37.4	172.7 ± 40.1	0.309	0.126
LDL (mg/dL, mean ± SD)	93.1 ± 31.8	91.5 ± 30.9	93.2 ± 32.4	94.4 ± 31.9	0.396	0.176
^c^HDL (mg/dL, mean ± SD)	47.7 ± 14.6	47.4 ± 14.3	47.7 ± 13.6	48.9 ± 15.9	0.816	0.527
^d^Triglyceride (mg/dL, mean ± SD)	159.2 ± 95.7	157.0 ± 89.4	157.7 ± 85.7	162.8 ± 110.3	0.607	0.361
^e^25-OH vitamin D (ng/mL, mean ± SD)	18.5 ± 8.5	19.1 ± 9.0	18.5 ± 7.3	17.9 ± 9.2	0.126	0.042
^f^24 hour urine sodium (mmol/day, mean ± SD)	154.8 ± 67.7	156.4 ± 64.7	155.5 ± 70.2	152.7 ± 67.9	0.44	0.437

*Abbreviations*: *SD* standard deviation, *eGFR* estimated glomerular filtration rate, *BMI* body mass index, *CKD* chronic kidney disease, *ESA* erythropoiesis-stimulating agent, *LDL* low density lipoprotein, *HDL* high density lipoprotein

^a^Kruskal-Wallis test was used to evaluate *P* value

^b^Hemoglobin A1c was measured only in the diabetic patients. (Total 724 patients; college or beyond group, 205 patients; completed high school 242 patients; lower than high school 277 patients)

^c^HDL was measured in 1342 patients (missing value = 19)

^d^Triglyceride was measured in 1329 patients (missing value = 32)

^e^25-OH vitamin D was measured in 1331 patients (missing value = 30)

^f^24 hour urine sodium was measured in 1240 patients (missing value = 121)

### Comparison of baseline characteristics according to socioeconomic status

We compared baseline characteristics according to educational attainment (Table 1) and monthly income levels (Table 2). With respect to educational attainment strata, the mean age was 2 years older in the lowest educational group. Subjects in the lowest education group exhibited the lowest eGFR (47.1 ± 23.3 for ‘college or beyond,’ 46.7 ± 26.6 for ‘completed high school,’ and 42.7 ± 25.0 mL/min/1.73 m^2^ for ‘less than high school’ groups, respectively; *P* for trend = 0.008). The higher education group was associated with higher monthly family income (*P* for trend < 0.001). The prevalence of anemia increased with decreasing level of educational attainment (41.2%, 48.8%, and 57.0%, respectively; *P* for trend < 0.001). The lowest educational attainment was an independent risk factor for anemia, even after adjusting for age, sex, and eGFR (‘less than high school’, OR 1.515, 95% CI 1.075–2.136, *P* = 0.018). The proportion of diabetic patients was the highest in the lowest education group. No group differences were exhibited in sodium excretion from 24-h urine collection.

**Table 2:** Baseline Characteristics according to Monthly Income Level

N	Total	Monthly income			*P*	*P* for trend
		> $ 4500	$1500 to $4500	< $ 1500		
	1361	269	701	391		
Age (year, mean ± SD)	60.9 ± 6.9	59.0 ± 6.6	60.6 ± 6.9	62.8 ± 6.7	< 0.001	< 0.001
Male (N, %)	860 (63.2)	199 (74.0)	433 (61.8)	228 (58.3)	< 0.001	< 0.001
LVH (N, %)	413 (30.3)	60 (22.3)	205 (29.2)	148 (37.9)	< 0.001	< 0.001
eGFR (mL/min/1.73m^2^)	45.5 ± 25.1	49.9 ± 24.9	46.5 ± 25.2	40.5 ± 24.3	< 0.001	< 0.001
BMI (kg/m^2^, mean ± SD)	24.6 ± 3.1	24.6 ± 2.8	24.6 ± 3.2	24.7 ± 3.2	0.586	0.403
CKD stage (N, %)					< 0.001	< 0.001
Stage G1	112 (8.2)	27 (10.0)	60 (8.6)	25 (6.4)		
Stage G2	226 (16.6)	58 (21.6)	115 (16.4)	53 (13.6)		
Stage G3a	250 (18.4)	54 (20.1)	145 (20.7)	51 (13.0)		
Stage G3b	322 (23.7)	60 (22.3)	171 (24.4)	91 (23.3)		
Stage G4	352 (25.9)	59 (21.9)	162 (23.1)	131 (33.5)		
Stage G5	99 (7.3)	11 (4.1)	48 (6.8)	40 (10.2)		
Underlying renal disease (N, %)					< 0.001	< 0.001
Diabetic nephropathy	429 (31.8)	59 (22.0)	223 (32.2)	147 (27.8)		
Hypertensive nephropathy	327 (24.2)	71 (25.4)	173 (25.0)	83 (21.3)		
Glomerulonephritis	341 (25.3)	88 (32.8)	179 (25.8)	74 (19.0)		
Polycystic kidney disease	140 (10.4)	39 (14.6)	63 (9.1)	38 (9.8)		
Diabetes mellitus (N, %)	571 (42.0)	88 (32.7)	291 (41.5)	192 (49.1)		
^b^Hemoglobin A1c (%, mean ± SD)	6.9 ± 1.4	6.8 ± 1.2	6.9 ± 1.3	7.0 ± 1.5	0.293	0.125
Hypertension (N, %)	1334 (98.0)	268 (99.6)	688 (98.1)	378 (96.7)	0.069	0.041
Systolic blood pressure (mmHg)	128.84 ± 16.7	127.0 ± 14.1	128.3 ± 17.3	131.0 ± 17.1	0.005^a^	0.001
Diastolic blood pressure (mmHg)	75.9 ± 11.2	77.1 ± 10.2	75.6 ± 11.3	75.8 ± 11.5	0.175	0.224
Educational attainment (N, %)					< 0.001	< 0.001
College graduate or higher	437 (32.1)	163 (60.6)	214 (30.5)	60 (15.3)		
Completed high school	467 (34.3)	73 (27.1)	279 (39.8)	115 (29.4)		
Lower than high school	347 (33.6)	33 (12.3)	208 (29.7)	216 (55.2)		
Physical activity (N, %)					< 0.001	< 0.001
High	287 (21.1)	55 (20.4)	156 (22.3)	76 (19.4)		
Moderate	536 (39.4)	137 (50.9)	259 (36.9)	140 (35.8)		
Low	538 (39.5)	77 (28.6)	286 (40.8)	175 (44.8)		
Anemia (N, %)	662 (49.1)	101 (37.8)	345 (49.8)	216 (55.8)	< 0.001	< 0.001
ESA (N, %)	119 (8.7)	12 (4.5)	71 (10.1)	36 (9.2)	0.019	0.01
Serum laboratory values						
Hemoglobin (g/dL)	12.6 ± 2.0	13.2 ± 1.9	12.6 ± 2.0	12.2 ± 1.9	< 0.001	< 0.001
Creatinine (mg/dL)	1.9 ± 1.1	1.8 ± 1.0	1.9 ± 1.1	2.1 ± 1.2	< 0.001^a^	< 0.001
Albumin (g/dL)	4.2 ± 0.4	4.2 ± 0.3	4.1 ± 0.4	4.1 ± 0.4	0.003^a^	0.001
Calcium (mg/dL)	9.1 ± 0.5	9.2 ± 0.5	9.1 ± 0.5	9.1 ± 0.5	0.012	0.004
Phosphorus (mg/dL)	3.7 ± 0.7	3.6 ± 0.5	3.7 ± 0.7	3.8 ± 0.7	0.003^a^	0.001
Total cholesterol (mg/dL)	170.7 ± 38.2	171.0 ± 37.0	171.4 ± 39.4	159.3 ± 36.8	0.692^a^	0.508
LDL (mg/dL)	93.1 ± 31.8	91.1 ± 32.4	94.4 ± 31.9	92.1 ± 31.1	0.281	0.851
^c^HDL (mg/dL)	47.7 ± 14.6	49.7 ± 14.5	47.4 ± 14.2	46.9 ± 14.3	0.051	0.029
^d^Triglyceride (mg/dL)	159.2 ± 95.7	143.7 ± 101.3	160.6 ± 100.0	159.6 ± 83.4	0.692	0.585
^e^25-OH vitamin D (ng/mL)	18.5 ± 8.5	19.4 ± 8.2	18.7 ± 9.0	17.6 ± 7.9	0.019	0.006
^f^24 hour urine sodium (mmol/day)	154.8 ± 67.7	157.4 ± 67.5	152.1 ± 66.5	157.8 ± 69.6	0.348	0.797

*Abbreviations*: *SD* standard deviation, *eGFR* estimated glomerular filtration rate, *BMI* body mass index, *CKD* chronic kidney disease, *ESA* erythropoiesis-stimulating agent, *LDL* low density lipoprotein, *HDL* high density lipoprotein

^a^Kruskal-Wallis test was used to evaluate *P* value

^b^Hemoglobin A1c was measured only in the diabetic patients. (Total 724 patients; >$4500, 115 patients; $1500 to $4500, 152 patients; <$1500, 252 patients)

^c^HDL was measured in 1342 patients (missing value = 19)

^d^Triglyceride was measured in 1329 patients (missing value = 32)

^e^25-OH vitamin D was measured in 1331 patients (missing value = 30)

^f^24 hour urine sodium was measured in 1240 patients (missing value = 121)

When the subjects were categorized based on monthly income level, similar trends were observed in terms of age, eGFR, DM, anemia and 24-h sodium excretion (Table 2).

### Socioeconomic status and left ventricular hypertrophy

**Fig. 2:**
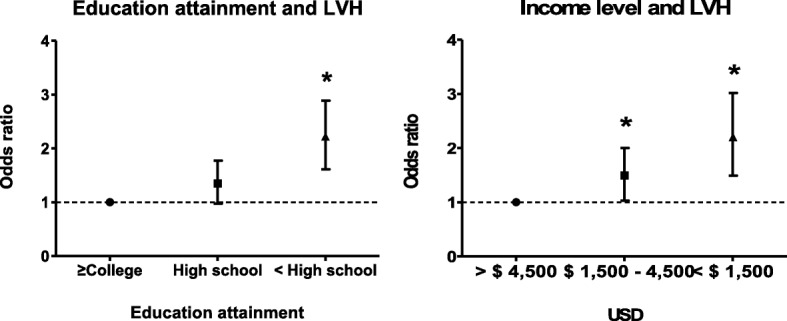
Odds Ratio for LVH according to SES in Univariate Analysis. Abbreviations: LVH, left ventricular hypertrophy; SES, socioeconomic status; USD, US dollar. *: *P* < 0.005

**Fig. 3:**
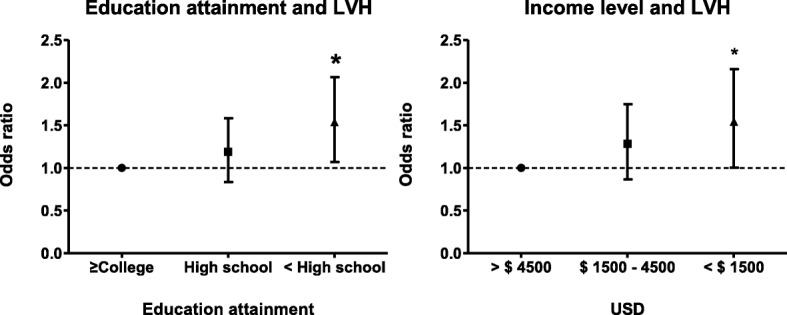
Odds Ratio for LVH according to SES in Multivariate Analysis. Adjusted for age, sex, mean arterial pressure, diabetes, CKD stage, physical activity Abbreviations: LVH, left ventricular hypertrophy; SES, socioeconomic status; CKD, chronic kidney disease; USD, US dollar. *: P < 0.005

The total number of patients diagnosed with LVH on echocardiography was 413 (30.3%). With the increase of the household income level or with the increase of the educational level, the prevalence of LVH gradually increased (Tables 1 and 2, *P* for trend < 0.001, respectively). In unadjusted analyses, risk of LVH increased with decreasing levels of educational attainment and monthly income level, respectively (Table 3, Table 4, and Fig. 2). In particular, the lowest educational level (lower than high school) was independently associated with LVH after adjusting for age, sex, body mass index, mean arterial pressure, DM, CKD stage and physical activity (‘college or beyond,’ reference; ‘completed high school’, OR 1.150, 95% CI 0.834–1.584; ‘less than high school’ OR 1.485, 95% CI 1.069–2.063; Fig. 3). Additionally, when anemia included as a covariate in the multi-variable model, the association between the lowest education level and LVH still showed the statistical significance (OR 1.454, 95% CI 1.042–2.028; *P* = 0.028, Table 3). Monthly income level is a risk factor for LVH in univariate and multivariate analysis adjusted age, sex, body mass index, mean arterial pressure, diabetes, CKD stages and physical activity. However, the inclusion of anemia as a covariates in the multivariable analysis attenuated the relationship between income level and LVH, as shown in the Table 4 (> $ 4500, reference; $ 1500 - 4500, OR 1.174, 95% CI 0.825–1.672, *P* = 0.373; < $ 1500, OR 1.415, 95% CI 0.962–2.081, *P* = 0.078).

**Table 3:** The relationship between LVH and educational attainment

	Univariate analysis		Multivariate (model 1)		Multivariate (model 2)	
	OR (95% CI)	*P*	OR (95% CI)	*P*	OR (95% CI)	*P*
Age						
50–59	reference		reference		reference	
60–69	1.575 (1.218–2.036)	0.001	1.409 (1.068–1.859)	0.015	1.442 (1.090–1.908)	0.01
≥ 70	2.468 (1.764–3.453)	< 0.001	2.246 (1.553–3.248)	< 0.001	2.308 (1.593–3.342)	< 0.001
Sex (vs. men)	1.797 (1.419–2.275)	< 0.001	1.863 (1.413–2.456)	< 0.001	1.885 (1.426–2.491)	< 0.001
BMI (reference ≤ 23.0)	2.254 (1.707–2.976)	< 0.001	2.401 (1.786–3.277)	< 0.001	2.457 (1.820–3.315)	< 0.001
MAP	1.018 (1.010–1.027)	< 0.001	1.018 (1.008–1.027)	< 0.001	1.019 (1.009–1.028)	< 0.001
DM	1.598 (1.266–2.017)	< 0.001	1.208 (0.934–1.563)	0.151	1.159 (0.890–1.510)	0.275
CKD stage						
1	reference		reference		reference	
2	1.409 (0.794–2.500)	0.241	1.464 (0.808–2.652)	0.209	1.365 (0.748–2.490)	0.311
3a	1.297 (0.735–2.291)	0.369	1.293 (0.713–2.343)	0.398	1.216 (0.666–2.223)	0.524
3b	1.983 (1.157–3.400)	0.013	1.776 (1.011–3.121)	0.046	1.613 (0.905–2.875)	0.105
4	3.185 (1.878–5.401)	< 0.001	3.007 (1.723–5.248)	< 0.001	2.609 (1.443–4.715)	0.002
5	3.680 (1.969–6.877)	< 0.001	3.146 (1.630–6.071)	0.001	2.645 (1.311–5.337)	0.007
Physical activity						
Low	0.942 (0.688–1.290)	0.711	0.786 (0.560–1.101)	0.161	0.817 (0.580–1.149)	0.245
Moderate	1.108 (0.812–1.511)	0.518	0.747 (0.532–1.049)	0.093	0.764 (0.541–1.078)	0.125
High	reference		reference		reference	
Anemia	1.790 (1.414–2.266)	< 0.001			1.219 (0.904–1.645)	0.194
Educational attainment						
College graduate or more	reference		reference		reference	
Completed high school	1.311 (0.971–1.770)	0.077	1.150 (0.834–1.584)	0.394	1.141 (0.825–1.579)	0.425
lower than high school	2.162 (1.616–2.892)	< 0.001	1.485 (1.069–2.063)	0.018	1.454 (1.042–2.028)	0.028

*Abbreviations*: *CI* confidential interval, *vs*., versus, *BMI* body mass index, *MAP* mean arterial pressure, *DM* diabetes mellitus, *CKD* Chronic kidney disease;

Model 1: age, sex, BMI, MAP, DM, CKD stage, physical activity, educational attainment, P for trend according to education attainment = 0.017, *P* for Hosmer and Lemeshow goodness of fit test = 0.090

Model 2: age, sex, BMI, MAP, DM, CKD stage, physical activity, anemia, educational attainment, P for trend according to education attainment = 0.025, *P* for Hosmer and Lemeshow goodness of fit test = 0.086

**Table 4:** The relationship between LVH and household income level

	Univariate analysis		Multivariate (model 3)		Multivariate (model 4)	
	OR (95% CI)	*P*	OR (95% CI)	*P*	OR (95% CI)	*P*
Age						
50–59	reference		reference		reference	
60–69	1.575 (1.218–2.036)	0.001	1.409 (1.067–1.861)	0.016	1.444 (1.091–1.913)	0.01
≥ 70	2.468 (1.764–3.453)	< 0.001	2.255 (1.561–3.260)	< 0.001	2.318 (1.601–3.356)	< 0.001
Sex (vs. men)	1.797 (1.419–2.275)	< 0.001	2.027 (1.559–2.635)	< 0.001	2.044 (1.569–2.664)	< 0.001
BMI (reference ≤ 23.0)	2.254 (1.707–2.976)	< 0.001	2.438 (1.813–3.278)	< 0.001	2.496 (1.849–3.369)	< 0.001
MAP	1.018 (1.010–1.027)	< 0.001	1.017 (1.008–1.027)	< 0.001	1.018 (1.009–1.028)	< 0.001
DM	1.598 (1.266–2.017)	< 0.001	1.212 (0.837–1.568)	0.143	1.159 (0.890–1.510)	0.275
CKD stage						
1	reference		reference		reference	
2	1.409 (0.794–2.500)	0.241	1.443 (0.798–2.611)	0.225	1.346 (0.739–2.451)	0.332
3a	1.297 (0.735–2.291)	0.369	1.254 (0.693–2.270)	0.454	1.178 (0.646–2.148)	0.592
3b	1.983 (1.157–3.400)	0.013	1.770 (1.008–3.108)	0.047	1.599 (0.898–2.847)	0.111
4	3.185 (1.878–5.401)	< 0.001	2.945 (1.689–5.136)	< 0.001	2.527 (1.399–4.565)	0.002
5	3.680 (1.969–6.877)	< 0.001	3.034 (1.572–5.856)	0.001	2.525 (1.252–5.091)	0.01
Physical activity						
Low	0.942 (0.688–1.290)	0.711	0.791 (0.565–1.109)	0.174	0.819 (0.582–1.152)	0.251
Moderate	1.108 (0.812–1.511)	0.518	0.747 (0.532–1.409)	0.092	0.762 (0.540–1.076)	0.122
High	reference		reference		reference	
Anemia	1.790 (1.414–2.266)	< 0.001			1.240 (0.920–1.672)	0.158
Income level						
> $ 4500	reference		reference		reference	
$ 1500-4500	1.440 (1.035–2.003)	0.03	1.230 (0.866–1.748)	0.247	1.174 (0.825–1.672)	0.373
< $ 1500	2.122 (1.491–3.018)	< 0.001	1.471 (1.002–2.158)	0.049	1.415 (0.962–2.081)	0.078

*Abbreviations*: *CI* confidential interval, *vs*., versus, *BMI* body mass index, *MAP* mean arterial pressure, *DM* diabetes mellitus, *CKD* Chronic kidney disease;

Model 3: age, sex, BMI, MAP, DM, CKD stage, physical activity, income level, *P* for trend according to income level = 0.045, *P* for Hosmer and Lemeshow goodness of fit test = 0.381

Model 4: age, sex, BMI, MAP, DM, CKD stage, physical activity, anemia, income level, *P* for trend according to income level = 0.06, *P* for Hosmer and Lemeshow goodness of fit test = 0.111

## Discussion

We performed cross-sectional analysis for the relationships between SES and LVH in a CKD population over 50 years of age. LVH was associated with parameters of SES, such as educational attainment and monthly income level, and these associations had graded responses. Additional analyses were conducted to clarify the association between SES and LVH, adjusting for body mass index, sex, age, blood pressure, DM, CKD stages and physical activity. After adjustment, lower education and income level remained a statistically significant determinant of LVH.

Compared with the statistical data of the Organization for Economic Co-operation and Development (OECD) statistics, the proportions of college graduates or above among the subjects at 25–45 years of age, were 69% for Koreans and 42% for the average OECD countries, respectively, while among those at 55–64 years of age, they are 18% in Korea and 26% in OECD average [20, 21]. Namely, since most of the younger Korean population are college graduates, educational attainment is not a suitable factor for evaluating the SES among younger Korean CKD patients. In such a respect, the present study included only the subjects at the age of 50 or older.

Usually, SES is defined by education, employment, income and poverty, and these indicators could influence one’s access to medical care and social support through insurance, housing stability and quality, accessibility to healthy food and degree of stress [10]. Since numerous complex interactions between the selected social determinants of health exist, it is difficult to elucidate the mechanism for the association between SES and many clinical outcomes regarding the incidence, progression of disease and mortality. In particular, low educational attainment has indirect effects on one’s understanding of disease and medical treatment. Low income level could affect one’s ability to engage in healthy behavior and access to healthcare services. It is important to identify clinical outcomes influenced by SES, because some determinants could be modified by government policies, social support for access medical care, and efforts of physicians and community members.

Explanations for the differences in the burden of LVH between various strata of socio-economic status remain speculative, but there are several potential reasons why lower SES could be an independent risk factor for LVH. First, socioeconomically disadvantaged patients tend to receive less vigorous treatment because of poorer access to medical care [22]. It is well known that early referral to a nephrologist improves clinical outcomes in CKD patients [23, 24] due to timely and proper management for prevention of disease progression. The primary system of South Korea health insurance system is the National Health Insurance (NHI), and nearly 96% of the total Korean population joined this NHI program [25]. It guarantees the basic medical cares including in-patient and out-patient health service, preventive care and prescription drugs, based on the co-payment system [26]. However, with the rapid expansion of enrolled population, the NHI has been under the heavy burdens due to increased medical expenditure [27]. Inevitably, the NHI program had to restrict the range of covered medical services at the possible level [28]. Therefore, some medical services or medical tests at high costs are not covered by the NHI, rendering them not easily accessible to the individuals at low SES. In addition, low SES leads to decreased understanding of treatment plans and poor compliance, which result in late diagnosis and disease progression.

Second, sympathetic stimulation is one of the mechanisms of LVH in low SES. The role of stress in health has been investigated since the 1950s [29] and the association between stress and cardiovascular disease is well known [30–33]. Sympathetic nervous system activity increases with various environmental factors, including low SES and stress. Lower SES is an important factor in psychosocial stress relative to higher SES [34]. It has been reported that chronic adrenergic stimulation can cause increased left ventricular mass [35]. Moreover, patients with CKD may be unable to adapt easily to stressful situations because stress hormones are metabolized and cleared by the kidney [36]. Patients with CKD may present inappropriate reactions to chronic stress. Thus, CKD patients with lower SES might be subjected both to more stress, and inappropriate responses to the stress.

Dietary differences between low and high SES groups are also related to LVH. One study reported that increased sodium retention might increase the risk of LVH by activation of the renin-angiotensin system and volume expansion [37]. Sodium intake tended to be higher among individuals with low SES [38–40]. In a study carried out among Chinese individuals, more educated participants had a lower intake of salt and soy sauce compared with less educated individuals [41]. This tendency might also be present in CKD populations, and dietary differences could contribute to a greater risk of LVH by sodium intake differences. Although this study did not collect dietary information, we attempted to evaluate sodium intake by measuring 24-h urine Na excretion, as it might reflect dietary sodium intake [42, 43]. However, our data did not show a significant difference in 24-h urine Na excretion with respect to educational attainment or income level. Since the main source of sodium intake in the western countries is processed foods (77% in the United States and 65–70% in the United Kingdom), people with low SES are less likely to have access to fresh food, thus, they are more likely to consume more processed food. In other words, the more processed food consumed, the more sodium intake. However, in South Korea, the main sources of salt intake are Kimchi, soup and stew, and these are easily accessible to anyone regardless of SES. Therefore, in Korea, personal salt intake depends more heavily on the personal salt preference, rather than on the SES of the individual. In addition, though adults should consume less than 2000 mg of sodium, or 5 g of salt per day according to guidelines issued by the World Health Organization (WHO) [44], Korean sodium intake is very high, because Korean food is generally very salty. In fact, looking at KNHANES data, the amount of sodium intake in Korea is 3669 mg, far exceeding 2000 mg [45].

Anemia has been shown to be an independent risk factor for LVH in CKD patients [46, 47]. The relationship between SES and anemia has been assessed primarily among adolescents and reproductive-aged women, for whom low SES is an important risk factor for anemia [48, 49]. Low iron intake among low SES populations also has been well established [50, 51]. In our subjects, anemia was more prevalent in the lowest educational group. Based on the above findings, we hypothesized that low SES might be associated with anemia, which, in turn, might lead to LVH. Our study showed that inclusion of anemia as a covariate in the multivariate analysis attenuated the significant association between low income and LVH. This means that anemia could partly be a contributing factor to the development of LVH in the CKD subjects in lower SES. However, even after adjustment for anemia as a covariate, low educational attainment still remained an independent risk factor for LVH (lower than high school, OR 1.454, 95% CI 1.042–2.028, *P* = 0.028; Table 3). Namely, other social determinants affected by SES but not included in the analysis might contribute to LVH. Further studies are warranted for elucidating the interconnected mechanism underlying the association between SES and LVH.

Our study tried to evaluate the economic status more accurately by surveying the individual or household monthly income, rather than an area income. However, relative to the association between education level and LVH, the association between monthly income level and LVH was somewhat attenuated after adjustment for anemia. It could be speculated that regular monthly income does not represent the overall economic status of a subject, particularly for an elderly or retired person with ample savings or real estate, but no regular monthly income. Therefore, a better parameter that could assess the economic status of an individual subject needs to be investigated.

This study is the first, to our knowledge, to elucidate that lower SES is an independent risk factor of LVH among CKD population. However, several limitations exist. First, although LVH is influenced by many factors, and we tried to adjust as many factors as possible related with health and dietary behavior, there still remains a possibility for a residual confounder. Since this study was conducted as a cross-sectional analysis, we could not determine causality between SES and LVH. However, longitudinal follow-up of the same study subjects will show us the causal relationship between SES and CV outcome. Second, we have excluded those who did not respond to the self-questionnaire and LV mass measurement. Because these patients are more likely to have poor compliance, it might influence the results. In addition, because information on the income and educational status was based only on the self-report, there may be a reporting bias. Although echocardiography was performed at each of nine participating centers, the data coordinating center of the KNOW-CKD Study collected each measurement parameter, calculated LV mass index, and relative wall thickness and classified LV geometry following a uniform criteria from the American Society of Echocardiography [17]. Lastly, the study enrolled only ethnic Korean patients; thus, it cannot provide information on the ethnic disparities in CKD.

## Conclusions

In summary, we identified a novel relationship between SES and LVH in CKD patients. Lower SES, defined by educational attainment and monthly income level, is an independent factor for LVH among CKD patients. Further studies are needed to explore the causal relationships between the SES and adverse cardiovascular outcomes in the CKD population, to address factors related to socio-environmental causes of LVH and to develop preventive strategies for CV mortality in patients with kidney disease. Such efforts will minimize socio-economic disparities, and improve CV outcomes for patients with CKD.

## Abbreviations

CI: Confidential interval
CKD: Chronic kidney disease
CKD-EPI: Chronic Kindey Disease Epidemiology Collaboration
CV: Cardiovascular
DM: Diabetes mellitus
eGFR: Estimated glomerular filtration rate
ESRD: End-stage renal disease
HTN: Hypertension
KNHANES: Korea National Health and Nutrition Examination Survey
KNOW-CKD: KoreaN Cohort Study for Outcome in Patients With Chronic Kidney Disease
LV: Left ventricle
LVH: Left ventricular hypertrophy
LVIDd: Left ventricular internal diameter end diastole
MET: Metabolic equivalent of task
NHI: National Health Insurance
OR: Odds ratio
PWTd: Posterior wall thickness in diastole
SES: Socioeconomic status
SWTd: Septum wall thickness in diastole
WHO: World Health Organization
