# Correction to: The association between socioeconomic disparities and left ventricular hypertrophy in chronic kidney disease: results from the KoreaN Cohort Study for Outcomes in Patients With Chronic Kidney Disease (KNOW-CKD)

**DOI:** 10.1186/s12882-018-1020-4

**Published:** 2018-09-04

**Authors:** Eunjeong Kang, Joongyub Lee, Hyo Jin Kim, Miyeun Han, Soo Wan Kim, Kyu-Beck Lee, Suah Sung, Tae-Hyun Yoo, Wookyung Chung, Curie Ahn, Kook-Hwan Oh

**Affiliations:** 1 Department of Internal Medicine, Seoul National University College of Medicine, Seoul, Korea; 2 School of Medicine, Inha University, Incheon, Korea; 3 Department of Prevention and Management, Inha University Hospital, Incheon, Korea; 4 Department of Internal Medicine, Dongguk University Gyeongju Hospital, Gyeongju, Korea; 5 Department of Internal Medicine, Pusan National University Hospital, Pusan, Korea; 6 Department of Internal Medicine, Chonnam National University Medical School, Gwangju, Korea; 7 Division of Nephrology, Kangbuk Samsung Hospital, Sungkyunkwan University School of Medicine, Seoul, Korea; 8 Department of Internal Medicine, Nowon Eulji Medical Center, Eulji University, Seoul, Korea; 9 Department of Internal Medicine, Yonsei University College of Medicine, Seoul, Korea; 10 Department of Internal Medicine, Gachon University Gil Medical Center, Gachon University School of Medicine, Incheon, Korea

## Correction

Following publication of the original article [1], an error in one of the author names was reported. In this Correction the incorrect and correct author name is listed. The original article has been updated.

Incorrect author name upon publication:

- Woo Wan Kim

The correct author name:

- Soo Wan Kim

## References

[1] Kang E (2018). The association between socioeconomic disparities and left ventricular hypertrophy in chronic kidney disease: results from the KoreaN Cohort Study for Outcomes in Patients With Chronic Kidney Disease (KNOW-CKD). BMC Nephrol.

